# CyaA translocation across eukaryotic cell membranes

**DOI:** 10.3389/fmolb.2024.1359408

**Published:** 2024-03-22

**Authors:** Amiel Abettan, Minh-Ha Nguyen, Daniel Ladant, Luca Monticelli, Alexandre Chenal

**Affiliations:** ^1^ Institut Pasteur, Université de Paris Cité, CNRS UMR3528, Biochemistry of Macromolecular Interactions Unit, Paris, France; ^2^ Molecular Microbiology and Structural Biochemistry Laboratory, CNRS UMR 5086, University of Lyon, IBCP, Lyon, France; ^3^ Université de Paris Cité, Paris, France; ^4^ Institut Pasteur, Université de Paris Cité, CNRS UMR3528, Biological NMR and HDX-MS Technological Platform, Paris, France; ^5^ Institut National de la Santé et de la Recherche Médicale (INSERM), Lyon, France

**Keywords:** membrane protein, bacterial toxin, translocation across membranes, biological membrane, molecular modeling & simulation

## Introduction

The adenylate cyclase (CyaA) toxin is a major virulence factor produced by *Bordetella pertussis*, the causative agent of whooping cough and is involved in the early stages of respiratory tract colonization (Guiso, 2017; Novak et al., 2017). This toxin invades eukaryotic cells through a unique but poorly understood mechanism that involves a direct translocation of its N-terminal adenylate cyclase catalytic domain (ACD) across the host plasma membrane (Voegele et al., 2018a). ACD produces supraphysiological levels of cAMP (Karst et al., 2010; Ahmad et al., 2016; O'Brien et al., 2017; Skopova et al., 2017), leading to cell death (Sakamoto et al., 1992; Ladant and Ullmann, 1999; Vojtova et al., 2006; Novak et al., 2017). The study of the translocation mechanism proved to be a formidable challenge, as the protein most likely features different conformations during the different steps of the translocation process, and no high-resolution structure is available yet. In this Opinion paper, we summarize the progress made so far in understanding the translocation mechanism and discuss possible avenues to address open questions in the field.

## State of the art

CyaA is a 1706-residue long multi-domain protein (Ladant and Ullmann, 1999), consisting of five domains: (1) the adenylate cyclase domain (ACD, residues 1-364) which is activated by calmodulin (CaM) binding and produces cAMP at the expense of ATP (Guo et al., 2005; Karst et al., 2010; O'Brien et al., 2017); (2) the translocation region (TR, residues 365-527) which is implicated in ACD translocation into target cells (Karst et al., 2012); (3) the hydrophobic region (HR, residues 528-710) which is able to form pores into membranes (Benz, 2016); (4) the acylation region (AR, residues 711-1005) which contains two lysine residues, K860 and K983, that are post-translationally acylated by a dedicated *B. pertussis* acyltransferase CyaC (Barry et al., 1991; Hackett et al., 1994; Westrop et al., 1996; O'Brien et al., 2019); this modification is essential for proper refolding of CyaA (Karst et al., 2014) and ACD translocation *in vivo* and *in vitro* (Barry et al., 1991; Novak et al., 2017); (5) the cell-receptor binding domain (RD, residues 1006-1706), made up of approximately 40 copies of calcium-binding RTX (Repeat-in-Toxin) motifs (Linhartová et al., 2010; Alouf et al., 2015; O'Brien et al., 2015; Benz, 2016). The C-terminal extremity of RD contains a secretion signal recognized by the dedicated type 1 secretion system (T1SS), made of *B. pertussis* CyaB, CyaD, and CyaE proteins (Masure et al., 1990). The secretion process is a processive mechanism initiated from the C-terminus of the protein as it exits first from the T1SS machinery. Once secreted through the T1SS, the toxin binds calcium in the extracellular medium (Bauche et al., 2006; Sotomayor-Perez et al., 2015; O'Brien et al., 2018). A calcium-induced disorder-to-order transition of RD occurs upon CyaA secretion from the low-calcium concentration of the bacterial cytosol to the calcium-rich extracellular environment (Rose et al., 1995; Bauche et al., 2006; Chenal et al., 2009; Chenal et al., 2010; Sotomayor Perez et al., 2010; Sotomayor-Pérez et al., 2011; Sotomayor-Pérez et al., 2013; O'Brien et al., 2015; Bumba et al., 2016; O'Brien et al., 2018). CyaA folding is an acylation-dependent and calcium-driven sequential process (Karst et al., 2014; Cannella et al., 2017; O'Brien et al., 2019).

**FIGURE 1:**
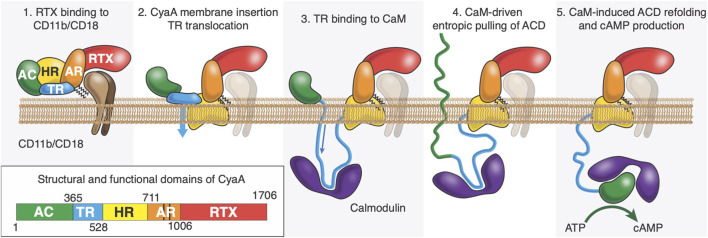
After its synthesis in the bacteria, CyaA is secreted through the Type-1 secretion system (T1SS). Once refolded in the extracellular space, CyaA interacts with CD11b/CD18 receptor via its RTX domain (Panel 1). CyaA may also directly interact with the plasma membrane of the host. It is proposed that the HR would interact with the host membrane (Panel 2) and TR would cross it (flip event) while binding with high affinity to Calmodulin, an abundant protein in eukaryotic cells (Panel 3). We also hypothesize that the complex between P454 and CaM favors the translocation of ACD by exerting an entropic pulling effect that induces the unfolding of ACD (Panel 4). Once in the cytoplasm, ACD refolds upon CaM binding (O’Brien et al., 2017), which activates the enzymatic activity of ACD, leading to overproduction of cAMP*.*

Intoxication of target cells occurs via a unique process among known bacterial toxins. CyaA can intoxicate target cells via a high affinity interaction between RD and the CD11b/C18 integrin that is expressed by a subset of leukocytes (Guermonprez et al., 2001; Guermonprez et al., 2002; El-Azami-El-Idrissi et al., 2003; Masin et al., 2005; Goldsmith et al., 2022) (neutrophils, dendritic cells (DC) and macrophages). CyaA can also invades host cells, such as epithelial cells (Angely et al., 2017; Angely et al., 2020), in the absence of CD11b/CD18 receptors by a direct interaction with the plasma membrane of eukaryotic cells; this process can be advantageously analyzed on mammalian erythrocytes as these cells are devoid of any membrane trafficking (Guermonprez et al., 2001; Guermonprez et al., 2002; El-Azami-El-Idrissi et al., 2003; Masin et al., 2005; Fiser et al., 2007; Chenal and Ladant, 2018). CyaA membrane translocation is calmodulin dependent (Voegele et al., 2021), calcium dependent, membrane-potential dependent and requires acylation on the lysine residues K860 and K983 located in AR (Fiser et al., 2007; Bumba et al., 2010; Karst et al., 2012; Uribe et al., 2013; Alouf et al., 2015). We propose that ACD is translocated across the plasma membrane into the cytoplasm through a transient and local membrane destabilization mediated by TR (Subrini et al., 2013; Voegele et al., 2018b; Voegele et al., 2021) (see Figure 1). Once translocated in the cytoplasm, TR interacts with CaM, and this high affinity TR:CaM complex exerts an entropic pulling effect on ACD located in the extracellular environment (Voegele et al., 2021). We hypothesize that the TR:CaM entropic pulling effect induces the unfolding of ACD followed by its translocation across the plasma membrane (Voegele et al., 2021). Calmidazolium, a potent CaM inhibitor, prevents CaM:TR complex formation and subsequently ACD translocation (Voegele et al., 2021; Léger et al., 2022). Once in the cytoplasm, ACD refolds upon CaM binding (Karst et al., 2010; O'Brien et al., 2017), which activates the enzymatic activity of ACD, leading to overproduction of cAMP and cell death.

In an effort to understand the determinants of protein translocation, we identified a peptide, corresponding to residues 454 to 485 of CyaA, coined P454, that exhibits membrane-active properties and interacts with high affinity with calmodulin. P454 forms a helix upon interaction with membranes and exhibits membrane-active properties (Subrini et al., 2013): not only it binds to membranes, but it also permeabilizes lipid vesicles. These experimental observations were supported by molecular dynamics simulations (Voegele et al., 2018b). Most importantly, deletion of the P454 segment abolishes CyaA intoxication of target cells (Karst et al., 2012).

How does P454 contribute to the transport of ACD across the lipid membranes into the cytosol? We hypothesized that in an early step of the translocation process, the P454 segment in CyaA can destabilize the cell membrane and translocate across the lipid bilayer to reach the cytosolic compartment, where it can bind the intracellular CaM; CaM binding would then drag the polypeptide chain inside cell via an entropic pooling force (Voegele et al., 2021). The structure and folding state of ACD before and upon membrane translocation (i.e., in the absence of CaM) is unknown yet, however, we showed that ACD is weakly stable in solution, a state favorable for its translocation across the plasma membrane (Karst et al., 2010; O'Brien et al., 2017; Voegele et al., 2018b).

One powerful method to investigate peptide translocation relies on the use of droplet interface bilayers (DIB) (Bayley et al., 2008). Briefly, two populations of droplets containing a buffer solution are delimited by a phospholipid monolayer, surrounded by a hydrophobic solvent; one population (*cis* droplets) contains a dye-labelled (fluorescent) peptide, while the other population (*trans* droplet) contains holo-CaM. When the lipid monolayer of the *cis* droplet contacts the lipid monolayer of the *trans* droplet, a lipid bilayer is formed at the interface. The transfer of fluorescence from the *cis* (fluorescent) droplet to the *trans* (non-fluorescent) droplet across the lipid bilayer interface can be measured. We performed the experiment with P454, and observed fluorescent rings staining the *cis* droplets at the beginning of the experiment, indicating that the peptides interact with the cis lipid monolayer. After a few minutes, fluorescence was detected within the *trans* compartment, indicating translocation of P454 and P454:CaM complex formation (Voegele et al., 2021). In the context of the full length CyaA toxin, we proposed that the CaM/P454 complex provides the energy required to overcome the penalty of ACD translocation across the plasma membrane by an entropic pulling effect (Voegele et al., 2021).

Questions regarding the role of membrane composition in peptide/membrane interaction and peptide translocation are very relevant for a mechanistic understanding of ACD translocation across plasma membrane. Experiments show that P454 binding depends on the lipid headgroup charge, acyl chain fluidity (i.e., acyl chain length and degree of saturation) and lipid polymorphism (Voegele et al., 2017; Voegele et al., 2018b). Unsaturated and negatively charged lipids promote P454 membrane partitioning, while negative membrane curvature and increased membrane rigidity (i.e., saturated acyl chains and cholesterol) do the opposite (Voegele et al., 2018b). The affinity of P454 for negatively charged membranes may be explained by the presence of two arginine residues, R461 and R474. Both arginine residues from P454 contribute to membrane active properties and calmodulin affinity; there is a correlation between the charge, the permeabilization efficiency, the free energy of solution-to-membrane partitioning and CaM binding (Voegele et al., 2018b). Substitutions of arginine by glutamate residues strongly reduce the membrane active properties and CaM affinity for P454 (Voegele et al., 2021). CyaA mutants harboring these mutations (R to E) still binds to the plasma membrane but the translocation of ACD is completely abolished.

Based on available data, we proposed a model of CyaA translocation into eukaryotic cells (Figure 1). We propose that, once TR is translocated into the cytoplasm, P454:CaM binding with high affinity induces unfolding of ACD. The disordered state of ACD would favor its translocation across membrane. The free energy gain due to the formation of the P454:CaM complex would effectively pull the unfolded ACD domain across the membrane. However, this hypothesis requires translocation of TR across the host membrane, which has been verified only for the isolated P454 peptide on model membranes using DIBs. We hypothesize that the translocation of TR across the plasma membrane requires membrane potential and calcium influx induced by CyaA membrane insertion.

## Discussion

The mechanism of protein translocation illustrated in Figure 1 is supported by experimental evidence (Voegele et al., 2021), but its details are not established, and some questions remain open. How does the entropic pulling work? What are the sequence determinants for the translocation of the P454 peptide? Does translocation across the host membrane require partial or full unfolding of ACD? Overall, in our view, a better understanding of CyaA translocation requires a quantitative, molecular-level description of the series of events from CyaA secretion to ACD translocation.

First of all, a better knowledge of CyaA structure and dynamics will greatly contribute to understanding protein translocation. We expect that a combination of cryo-EM, SEC-SAXS, bioinformatics, and computer simulations will be necessary to describe the conformational preferences and the dynamics of CyaA. Novel structure prediction algorithms based on machine learning, such as Alphafold (Jumper et al., 2021) (AF2), may contribute to a better understanding of protein structure; however, these algorithms are based on knowledge extracted from databases of existing protein structures, that is rather limited when it comes to membrane proteins. One possible workaround is to use AF2 only for protein regions known not to be inserted in the membrane, and physics-based models or experimental structural information for membrane-interacting fragments. As for experimental information, cryo-EM methods hold great promise, as their success is not limited to soluble proteins: a number of membrane protein structures have been resolved at high resolution in recent years (Renaud et al., 2018). As for computer simulations, we expect them to make a significant contribution to the study of both the structure and the dynamics of the system. Different scales relevant to the translocation process should be considered: simulations at the atomistic scale can be used to explore the conformational flexibility of relatively short peptides (such as P454) in different environments (i.e., the cytosol vs. the membrane interior) but remain prohibitively expensive for large systems (Gupta et al., 2022), such as the one including the entire CyaA protein, embedded in a membrane with complex (plasma membrane-like) lipid composition. They also remain too expensive to enable predictions on the actual dynamics of peptide translocation, that probably takes place on time scales of milliseconds or longer. On the other hand, coarse-grained (CG) simulations can be used to reach millisecond time scales (Gupta et al., 2022; Marrink et al., 2023) and to explore translocation dynamics and thermodynamics of peptides (Kabelka et al., 2021), particularly when combined to enhanced sampling techniques (Barducci et al., 2011), but are generally less reliable in the exploration of protein conformational changes (Monticelli et al., 2008; Periole et al., 2009; Borges-Araújo et al., 2023). This limitation also applies to the latest version of the Martini CG force field (Souza et al., 2021), but we expect that further development will improve the description of conformational flexibility in proteins (Borges-Araújo et al., 2023). The major strength of CG approaches remains speed, and can be harnessed by calculating translocation properties for series of mutant peptides, to clarify the molecular driving forces of the translocation process.

In general, differences between *in silico*, *in vitro,* and *in vivo* systems should be carefully considered, as results obtained in model systems cannot always be directly transferred to living systems. On one hand, in model systems (*in silico* and *in vitro*) it is easier to control and change system properties (composition, membrane potential, calcium concentration, etc.). On the other hand, simplifications contained in such model systems sometimes result in failure to reproduce results obtained in cells.

Another important aspect that deserves a more quantitative description is the partitioning of CyaA and its subdomains into membranes. Numerous methods are available for this, and we recently developed a label-free NMR method, B2LiVe, based on selective excitation of amide resonances, to quantify affinity of proteins and peptides for membranes (Anderluh, 2023; Sadi et al., 2023).

Besides protein binding, also protein translocation should be described more quantitatively, for instance by investigating the translocation energetics of TR and ACD, in the presence and in the absence of the CD11b/CD18 receptor. We are also interested to determine the contribution of the factors involved in the translocation process, *i.e.*, the membrane potential, the calcium gradient, the TR:CaM entropic pulling, and the contribution of lipid composition, polymorphism and charge, as well as membrane fluidity, rigidity, thickness and curvature. A number of different approaches are available to study translocation, for example, tethered bilayer membranes (Veneziano et al., 2013; Veneziano et al., 2017). This approach demonstrated that the translocation of the catalytic domain of CyaA is strictly dependent on the presence of CaM, calcium gradient and on the application of a negative trans-membrane potential (Veneziano et al., 2013), as found for entry into eukaryotic cells (Otero et al., 1995). However, this method is laborious and time consuming, and DIBs are emerging as a promising alternative (Bayley et al., 2008; Voegele et al., 2021).

Finally, questions on the molecular determinants of P454 binding to CaM and peptide translocation can be explored by studying mutants, both at the experimental and at the computational level. In perspective, we expect that mutants of P454 (or larger portions of the TR domain) could be optimized to design inactivated CyaA recombinant proteins with improved translocation capabilities. This would be particularly interesting for enhancing the antigen-delivery efficacy of recombinant CyaA-based vaccines (Fayolle et al., 1996; Karimova et al., 1998; Masin et al., 2005).
